# One-Pot Cu/TiO_2_ Nanoparticles Synthesis for Trans-Ferulic Acid Conversion into Vanillin

**DOI:** 10.3390/molecules24213985

**Published:** 2019-11-04

**Authors:** Paulette Gómez-López, Noelia Lázaro, Clemente G. Alvarado-Beltrán, Antonio Pineda, Alina M. Balu, Rafael Luque

**Affiliations:** 1 Departamento de Química Orgánica, Universidad de Cordoba, Campus de Rabanales, Edificio Marie Curie (C-3), Ctra Nnal IV-A, Km 396, E14014 Cordoba, Spain; gomezl.paulette@gmail.com (P.G.-L.); bt2laron@uco.es (N.L.); q82pipia@uco.es (A.P.); qo2balua@uco.es (A.M.B.); 2 Facultad de Ingeniería Mochis, Universidad Autónoma de Sinaloa, Fuente de Poseidón y Prol. Angel Flores, S.N., Los Mochis Sin. 81223, Mexico; 3 Scientific Center for Molecular Design and Synthesis of Innovative Compounds for the Medical Industry, People’s Friendship University of Russia (RUDN University), Moscow 117198, Russia

**Keywords:** one-pot synthesis, TiO_2_, Cu nanoparticles, vanillin, trans-ferulic acid, heterogeneous catalysis

## Abstract

In this study, the co-synthesis of TiO_2_ and Cu metallic nanoparticles obtained via one-pot cost-efficient hydrothermal process has been addressed. Different nanocatalysts with Cu contents were characterized by X-ray diffraction, nitrogen porosimetry, scanning electron microscopy, and transmission electron microscopy. The TiO_2_ and Cu metallic nanoparticles were synthesized with copper loading up to one (Cu/Ti atomic ratio). Synthesized catalysts exhibited pore sizes in the mesoporous range and high surface areas above 150 m^2^/g. The particle size for TiO_2_ presented a homogeneous distribution of approximately 8 nm, moreover, Cu nanoparticles varied from 12 to >100 nm depending on the metal loading. The nanostructured materials were successfully tested in the conversion of trans-ferulic acid into vanillin under sustainable conditions, achieving the best performance for 0.3 Cu/Ti atomic ratio (70% vanillin yield).

## 1. Introduction

Vanillin has been consolidated as one of the most valuable substances, popular flavoring and aroma, because of its widespread use and commercialization as an additive in the pharmaceutical, cosmetic, and food industries [1]. Only approximately 1% of the total worldwide production corresponds to natural extraction [2,3]. In this regard, the synthetic production of vanillin has been explored employing several raw materials such as guaiacol [4], isougenol [5], safrole, lignin [2], and ferulic acid [1,6,7,8,9,10]. In this sense, trans-ferulic acid is nontoxic and highly abundant in lignocellulosic biomass as lignin and pectin in seeds and leaves [11,12]. The conversion of biomass into a chemical has been the subject of intense research and one of the efforts has focused on the vanillin production from trans-ferulic acid. Recently, trans-ferulic acid biotransformation employing Escherichia coli [13] bacteria [14], microbial [1,15], fungal strain [16], enzymes [5,8], etc., has also been studied. Likewise, the use of metal oxide catalysts such as inorganic materials Bi_2_WO_6_ [17] or metalorganic frameworks [7,9,10] has been explored, even via photocatalytic conversion using TiO_2_ [18,19], as well as WO_3_-loaded TiO_2_ [20]. Biological transformations provide good vanillin yields but there are some problems related to the purification steps and the presence of remnant bacteria [13,15,20].

The chemical conversion by heterogeneous catalyst has been widely employed in industry. Metal oxides and metallic nanoparticles have been explored separately, but currently co-catalysts are widely studied [21,22]. For instance, metal nanoparticles have been supported on carbonaceous materials [23] and metal oxides [21]. Successful co-catalysis deposition has been reported and TiO_2_ is the most commonly used, due to its stability and high potential application on photocatalytic hydrogen generation [24] or CO_2_ reduction [21], among other attractive applications. Interestingly, TiO_2_ has been decorated with metals, especially Pt, Pd, Au, and Ag, which present great activity in hydrogenation [25], oxidation [26] and isomerization [27] reactions, however, such noble metals are quite expensive while Cu offers a cost-effective alternative in the catalysis field [28].

The synthesis of a co-catalyst, supported or decorated with metallic nanoparticles, has been processed in more than one step [22,23,24,25,26,27,28] employing high temperature or several solvents, surfactants, and precursors, some of which are toxic or highly harmful to the environment [23,24]. Previous works have reported that TiO_2_ was decorated by photodeposition [29], electrodeposition [28], microemulsion [24], chemical [30], and radiolytic reduction [31]. The one-pot synthesis offers a green chemistry and sustainable alternative necessary today.

In this work, we report for the first time the synthesis of Cu/TiO_2_ nanoparticles in one step via a solvothermal green process. Cu content has an impact in the synthesis, considering that high loading of such metal is necessary for the nanoparticles’ formation. We describe the application of Cu/TiO_2_ in the conversion of trans-ferulic acid. The influence of their morphology and structural characteristics in the high conversion of trans-ferulic acid into a vanillin are analyzed. Special attention has been paid to the particle size and its influence on catalytic performance.

## 2. Results and Discussion

**Figure 1:**
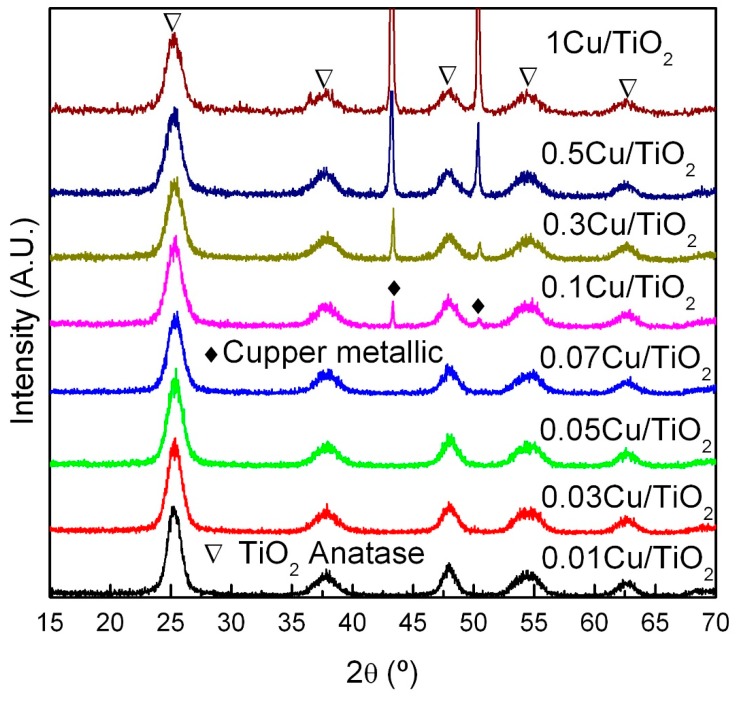
X-ray diffraction patterns corresponding to the Cu/TiO_2_ samples synthetized with different Cu content (TiO_2_, tetragonal and Cu, cubic structures).

X-ray diffraction (XRD) analysis for all samples are shown in Figure 1. The patterns confirm the presence of TiO_2_ for all samples in the anatase phase (PDF 21-1272, space group I 4_1_/amd). Moreover, the full width at half maximum for anatase peaks barely changed, maintaining the main value at approximatley 8.5 nm (± 0.5 nm) of crystallite size. For samples with atomic ratios higher than 0.1Cu/TiO_2_, the copper metallic cubic phase (PDF 04-0836, space group 225/Fm-3m) is present. The signal for the planes corresponding to (111) 43.29° and (200) 50.43° were found to be better defined at increased Cu content. The crystallite size for Cu/TiO_2_ ratios of 0.1, 0.3, 0.5, and 1 calculated using the Scherrer equation were 55, 64, 65, and 75 nm (± 2 nm), respectively. Therefore, XRD analysis confirmed the presence of the pure anatase for lower contents of Cu, in addition to the coexistence of TiO_2_ and copper metallic nanoparticles for copper content higher than 0.1Cu/TiO_2_. Interestingly, the unexpected presence of metallic Cu in the samples can be explained by the presence of a hydrogen donor solvent, ethanol, in the solvothermal process, which acts as a reducing agent [32].

**Figure 2:**
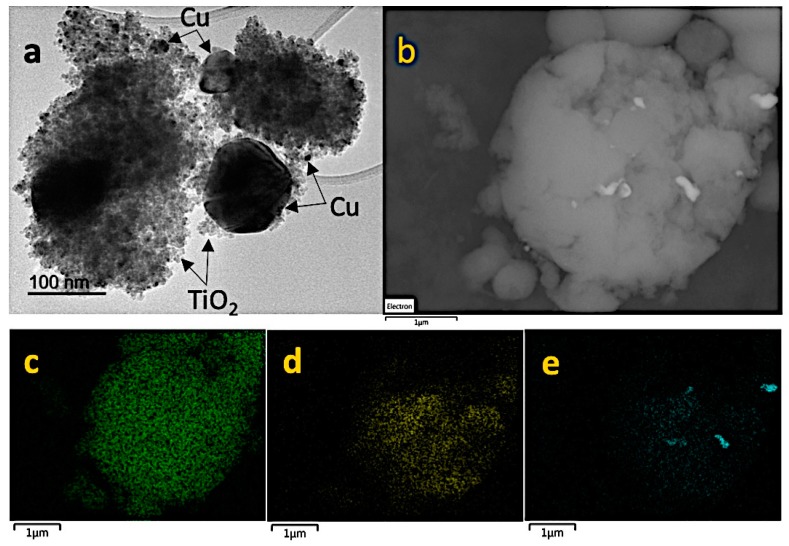
Micrographs of the 0.3Cu/TiO_2_ sample: (**a**) TEM image with TiO_2_ and Cu particles identified, (**b**) SEM image and EDX-mapping, (**c**) Ti, (**d**) O, and (**e**) Cu.

The morphology and the presence of TiO_2_ and Cu nanoparticles are confirmed by TEM and SEM micrographs (Figure 2). The TEM image in Figure 2a shows the presence of TiO_2_ nanoparticles homogeneously distributed, with a particle size of approximately 8 nm, and this information barely changes, independent of the Cu content. Moreover, the particle size distribution for Cu is not homogeneous, varying from 11 to >100 nm, without a preferential size. Cu nanoparticles could only be clearly seen when the Cu content was more than 0.1Cu/Ti atomic ratio. Furthermore, the SEM images (Figure 2b) and EDX-mapping micrographs of the 0.3Cu/TiO_2_ sample are shown in the Figure 2c–e for Ti, O, and Cu, respectively. The Ti and O are homogeneously distributed in the material. In addition, copper particles are also homogeneously well dispersed but with a clearly visible agglomeration. This is also observed for the samples with more Cu content (Figures S1 and S2).

The textural properties for most representative samples are shown on Table 1. The high surface area 254 m^2^/g, corresponds to the minimum content of copper (0.01Cu/TiO_2_), which is highly superior as compared with unmodified TiO_2_. There is an observable trend when the Cu contents increase, the surface area decreases, reaching 152 m^2^/g (see Figure S3), although the surface area is higher than other reports for Cu-TiO_2_ nanocomposites [24,31]. The pore diameter measured for mesoporous materials is approximately 4 nm for a low concentration of Cu. This value changes when the copper nanoparticles are present (Cu/Ti > 0.1), with values increasing to 6 nm, indicating that the agglomerated Cu particles at larger Cu loadings do not migrate to the pores (too large) and preferentially deposit on the external surface of titania, therefore, increasing the pore diameters in the material.

**Scheme 1:**
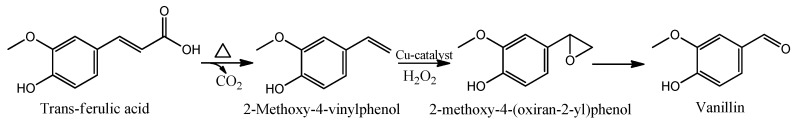
Transformation of trans-ferulic acid into vanillin, through the formation of 4-vinylguaiacol and 2-methoxy-4-propylphenol as intermediates.

The catalytic performance of the synthesized materials has been evaluated in the conversion of trans-ferulic acid into vanillin using hydrogen peroxide as an oxidant. Oxidation reactions constitute an important research area that allows important transformations among functional groups such as alcohols, aldehydes, ketones, and carboxylic groups. For the case investigated in this study, the transformation of trans-ferulic acid into vanillin, the reaction, as Scheme 1 illustrates, takes place in two steps. In the first step, trans-ferulic acid is thermally decarboxylated to form 4-vinylguaiacol or 2-methoxy-4-vinylphenol. Eventually, the use of a Cu-based catalyst together with a green oxidant (hydrogen peroxide) leads to the formation of vanillin. As an intermediate step, the double bond is oxidized to form the corresponding epoxide (not observed), which may be subsequently hydrolyzed to form the corresponding diol and finally, vanillin. In addition, the formation of 2-methoxy-4-propylphenol, a lignin model compound, has been detected by GC-MS. 

The decarboxylation reaction took place, already in blank runs (in the absence of catalyst), completely converting trans-ferulic acid into 2-methoxy-4-vinylphenol without detecting any additional products. For the catalytic tests, detected products were vanillin, 2-methoxy-4-vinylphenol, and 2-methoxy-4-propylphenol. Table 2 shows a summary of the most relevant results achieved by the catalyst, herein investigated. First, complete decarboxylation of trans-ferulic acid takes place (with the formation of minimum amounts of vanillin) when the reaction was performed in the presence of TiO_2_. Furthermore, the selectivity towards vanillin increases with Cu loading, with the highest observed for material 0.3Cu/TiO_2_ (Cu/Ti atomic ratio of 0.3). For the remaining materials with an atomic Cu/Ti loading higher than 0.3, the selectivity towards vanillin production decreases, related to an increase in particle size (64 vs. 75 nm for 0.3Cu/TiO_2_ and 0.1Cu/TiO_2_, respectively). There seems to be a trade-off between metal loading and particle size that makes 0.3Cu/TiO_2_ the most efficient material in the production of vanillin from trans-ferulic acid under the investigated conditions (30 min reaction, 60% selectivity to vanillin), that could be increased up to 70% after one hour. Unexpectedly, the products distribution remained unaltered once equilibrium was reached, although the oxidative conditions employed in the reaction can favor polymerization reactions. 

Our results are comparable with other results already reported in the literature. For instance, the use of Cu-MOF-74 reached a maximum vanillin yield of 70% after 4 h of reaction [9], significantly higher than the time required for the best of the catalysts in the trans-ferulic acid oxidation explored in this work. This fact could be attributed to the microporous nature of the metal-organic framework (MOF) materials, while the catalysts explored in our work present essentially a mesoporous nature, however, the yield achieved by 0.3Cu/TiO_2_ is still lower than that achieved by the porous coordination polymer HKUST-1 that afforded a vanillin yield of 95% after one hour [33]. Other approaches, such as electrocatalysis, have been shown to be less efficient in the production of vanillin from trans-ferulic acid reaching a catalytic performance sensitively lower than that reported by [10]. 

The catalysts could be recycled up to three to four reaction runs, however, with an associated decrease in selectivity to vanillin (maximum 35% to 40%). The decrease in selectivity seemed to be associated with Cu sintering and leaching (See Figure S4), (ca. 10–20 ppm Cu detected in solution by ICP-MS). 

## 3. Experimental Methods

### 3.1. Materials

Ninety-seven percent pure reagent grade titanium (IV) butoxide (Ti(OBu)_4_), 100% pure anhydrous ethanol (EtOH), and nitric acid 65% (HNO_3_) were purchased from Sigma-Aldrich (St. Luis MO, USA) and 99.5% pure copper (II) nitrate trihydrate (Cu(NO_3_)_2_ 3H_2_O), from Merck (Darmstadt, Hesse, Germany). The trans-ferulic acid (99% purity) and hydrogen peroxide solution 50 wt.% in water were acquired from Sigma-Aldrich (St. Luis, MO, USA) All the chemicals used in this work have been employed as received from the provider, without any additional purification steps. 

### 3.2. Synthesis of Cu/TiO_2_

The materials were prepared separately. The titanium and copper source solutions were prepared at room temperature as described here. First, titanium butoxide was added to ethanol and then nitric acid under continuous stirring during 30 min (1:30:1 molar ratio). At the same time, copper nitrate was dissolved in ethanol (1:15 molar ratio), stirring until it turns homogenously blue transparent. The Cu/Ti atomic ratio was calculated maintaining 50 mL of volume solution. Both solutions were subsequently mixed to obtain a blue transparent solution without precipitation, after 30 min the final solution was transferred into a steel-Teflon covered autoclave (100 mL of volume) and put into an oven at 200 °C for 6 h. Finally, the different materials were recovered as a powder by filtration and dried at room temperature for 24 h. Samples were denoted 0.01Cu/TiO_2_ for 1% copper with respect to Ti, and the copper content varied until 1Cu/TiO_2_ for Cu:Ti when the atomic ratio was 1:1.

### 3.3. Catalyst Characterization

The materials structure was evaluated by X-ray diffraction using a Bruker D8 Discover diffractometer (Bruker, Billerica, MA, USA) with Cu Kα radiation (λ = 1.54 Å). The XRD patterns were recorded in a 2θ scan range from 15° to 70° with a scan speed of 0.5 or 1 °/min. Phase identification and crystallite size fitted were carried out using Diffrac.suite Eva software, supported by the Power Diffraction File Database.

Textural properties were evaluated by nitrogen adsorption and desorption, and isotherm experiments were accomplished in a porosimeter Micromeritics ASAP 2000 instrument (Micromeritics, Norcross, GA, USA). Samples were previously degassed at 130 °C for 24 h under vacuum (*p* < 10^−2^ Pa). The specific surface area of the synthetized materials was calculated using the Brunauer, Emmett and Teller (BET) equation in the linear region (0.05 < Po < 0.22).

The TEM images were recorded in a JEOL JEM 1400 instrument (JEOL USA, Inc, Peabody, MA, USA) and assembled with a charge-coupled camera device (Zeiss, Oberkochen, Baden-Württemberg, Germany). Samples were previously suspended in ethanol and deposited on a copper grid. 

SEM-EDX micrographs were acquired in a JEOL-SEM JSM-7800 LV scanning microscope.

### 3.4. Catalytic Experiments

The catalytic evaluation of as-synthesized materials was carried out in the oxidation of trans-ferulic acid into vanillin using hydrogen peroxide as a green oxidant. In a typical experiment, 5 mmol of trans-ferulic acid, 1.2 mL H_2_O_2_ (50% *v/v* solution), 8 mL acetonitrile, and 0.1 g of catalyst at 90 °C were added in a glass tube using Radleys Parallel reaction station, withdrawing samples periodically. The samples were analyzed in a gas chromatograph Agilent Technologies 7890 A using a PetrocolTM DH (100 m × 0.25 mm × 0.50 µm) column and a flame ionization detector (FID). In addition, the identification of the different products was carried out using an Agilent 7820A GC/5977B High Efficiency Source (HES) MSD GC-MS (JEOL Ltd., Tokio, Japan).

## 4. Conclusions 

The Cu/TiO_2_ nanocatalysts, with different Cu/Ti atomic ratios in a range between 0.01 and 1, were prepared following a novel and sustainable solvothermal approach and Cu was obtained in the metallic phase due to the reducing environment employed during the materials preparation. 

The catalytic activity of the as-prepared materials was investigated in trans-ferulic acid transformation into vanillin. The catalytic activity of Cu/TiO_2_ materials in the investigated reaction was found to be influenced by two parameters, namely, metal loading and particle size, with 0.3Cu/TiO_2_ reaching maximum vanillin yields (ca. 70%).

## Figures and Tables

**Table 1:** Textural properties: Surface area (S_BET_), pore volume (V_BJH_), mesopore volume (V_meso_), and pore diameter (D_BJH_) obtained for selected Cu/TiO_2_ catalysts obtained by nitrogen physisorption measurements.

Catalyst	S_BET_ (m^2^/g)	V_BJH_ (cm^3^/g)	V_meso_ (cm^3^/g)	D_BJH_ (nm)
TiO_2_	143	0.13	0.10	3.5
0.01Cu/TiO_2_	254	0.25	0.23	3.9
0.05Cu/TiO_2_	191	0.22	0.19	4.8
0.1Cu/TiO_2_	204	0.32	0.27	6.0
0.3Cu/TiO_2_	173	0.30	0.26	6.0
0.5Cu/TiO_2_	152	0.28	0.26	6.4

**Table 2:** Catalytic activity of Cu/TiO_2_ materials in trans-ferulic acid oxidation to vanillin using hydrogen peroxide as green oxidant.

Catalyst	Conversion (%)	Selectivity (%)		
		2-Methoxy- 4-Vinylphenol	Vanillin	2-Methoxy- 4-Propylphenol
Blank	>99.9	>99.9	-	-
TiO_2_	>99.9	>94.2	5.7	-
0.01Cu/TiO_2_	>99.9	82.7	12.5	4.8
0.03Cu/TiO_2_	>99.9	75.5	19.3	5.2
0.05Cu/TiO_2_	>99.9	60.1	34.3	5.6
0.3Cu/TiO_2_	>99.9	36.1	60.7	3.2
1Cu/TiO_2_	>99.9	48.8	49.3	1.9

Reaction conditions: 0.1 g of catalyst; 5 mmol of trans-ferulic acid; 1.2 mL H_2_O_2_ (50% *v/v* solution); and 8 mL acetonitrile at 90 °C, 30 min reaction.

## Supplementary Materials

The Supplementary Material for this article can be found online at https://www.mdpi.com/1420-3049/24/21/3985/s1, Figure S1: SEM micrograph of 0.5Cu/TiO_2_, Figure S2: SEM micrograph of 1CuTiO_2_, Figure S3: N_2_ adsorption-desorption isotherm, Figure S4: DRX pattern for 0.5Cu/TiO_2_.
