# Supplementary material for: One-Pot Cu/TiO2 Nanoparticles Synthesis for Trans-Ferulic Acid Conversion into Vanillin
Source: Molecules. 2019 Nov 4;24(21):3985. doi: 10.3390/molecules24213985 (PMC6864876; doi:10.3390/molecules24213985)
Supplement: Supplementary file 1 [file molecules-24-03985-s001.pdf]

One-Pot Cu/TiO_2 Nanoparticles Synthesis for Trans-Ferulic Acid Conversion into Vanillin

Paulette Gómez-López[1], Noelia Lázaro[1], Antonio Romero[1], C.G. Alvarado-Beltrán[2*], Antonio Pineda[1], Alina M. Balu[1] and Rafael Luque,[1,3*]

[1] Grupo FQM-383, Departamento de Química Orgánica, Universidad de Cordoba, Cordoba, Spain

[2] Facultad de Ingeniería Mochis, Universidad Autónoma de Sinaloa, Fuente de Poseidón y Prol. Angel Flores, S.N., 81223, Los Mochis Sin., México

[3] Scientific Center for Molecular Design and Synthesis of Innovative Compounds for the Medical Industry, People's Friendship University of Russia (RUDN University), Moscow, Russia

Rafael Luque: q62alsor@uco.es , Clemente G. Alvarado Beltrán: calvarado@uas.mx

In this study, the co-synthesis of TiO_2 and Cu metallic nanoparticles obtained via one pot cost-efficient hydrothermal process has been addressed. The different nanocatalysts with different Cu content were characterized by X-ray diffraction, nitrogen porosimetry, scanning electron microscopy and transmission electron microscopy. The TiO_2 and Cu metallic nanoparticles were synthetized with a copper loading up to 1 (Cu/Ti atomic ratio). The synthesized catalysts present pore size in the mesoporous range and high surface areas above 150 m^2/g. The particles size for TiO_2 present homogeneous distribution around 8 nm, moreover, Cu nanoparticles varies from 12 to >100nm depending on the metal loading. The nanostructured materials were gratefully tested in the oxidation of trans-ferulic acid into vanillin under sustainable conditions. The materials were able to successfully convert the trans-ferulic acid into vanillin, achieving the best performance through the use of the TiO_2 catalyst with 0.3 Cu/Ti atomic ratio leading to a maximum vanillin yield of 70%.

KEYWORDS: **one-pot synthesis; TiO_2; Cu nanoparticles; vanillin; *trans-ferulic acid; heterogeneous catalysis.***

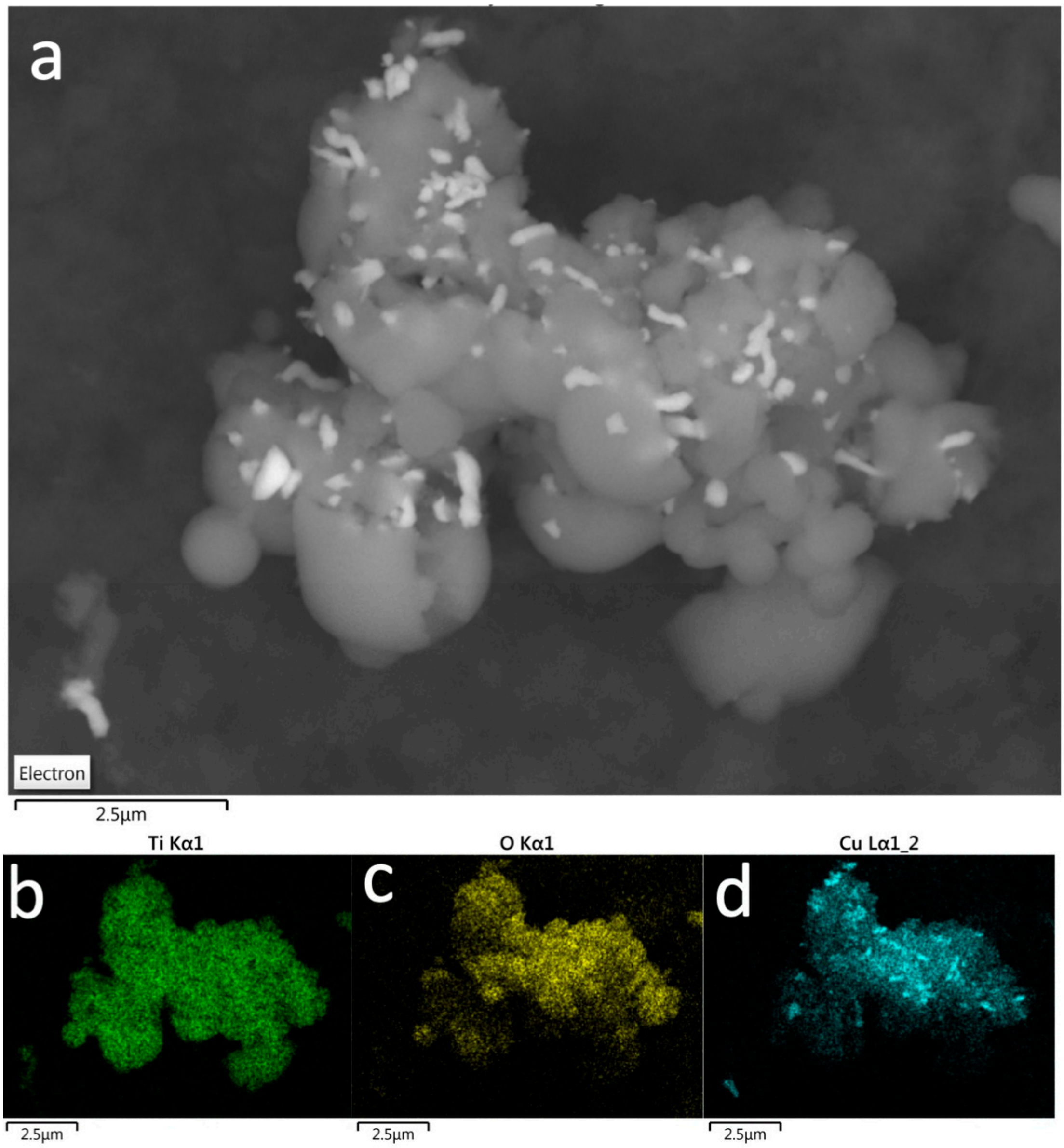

Figure S1. SEM micrograph of 0.5Cu/TiO_2 (a) and EDX-mapping (b) Ti, (c) O and (d) Cu

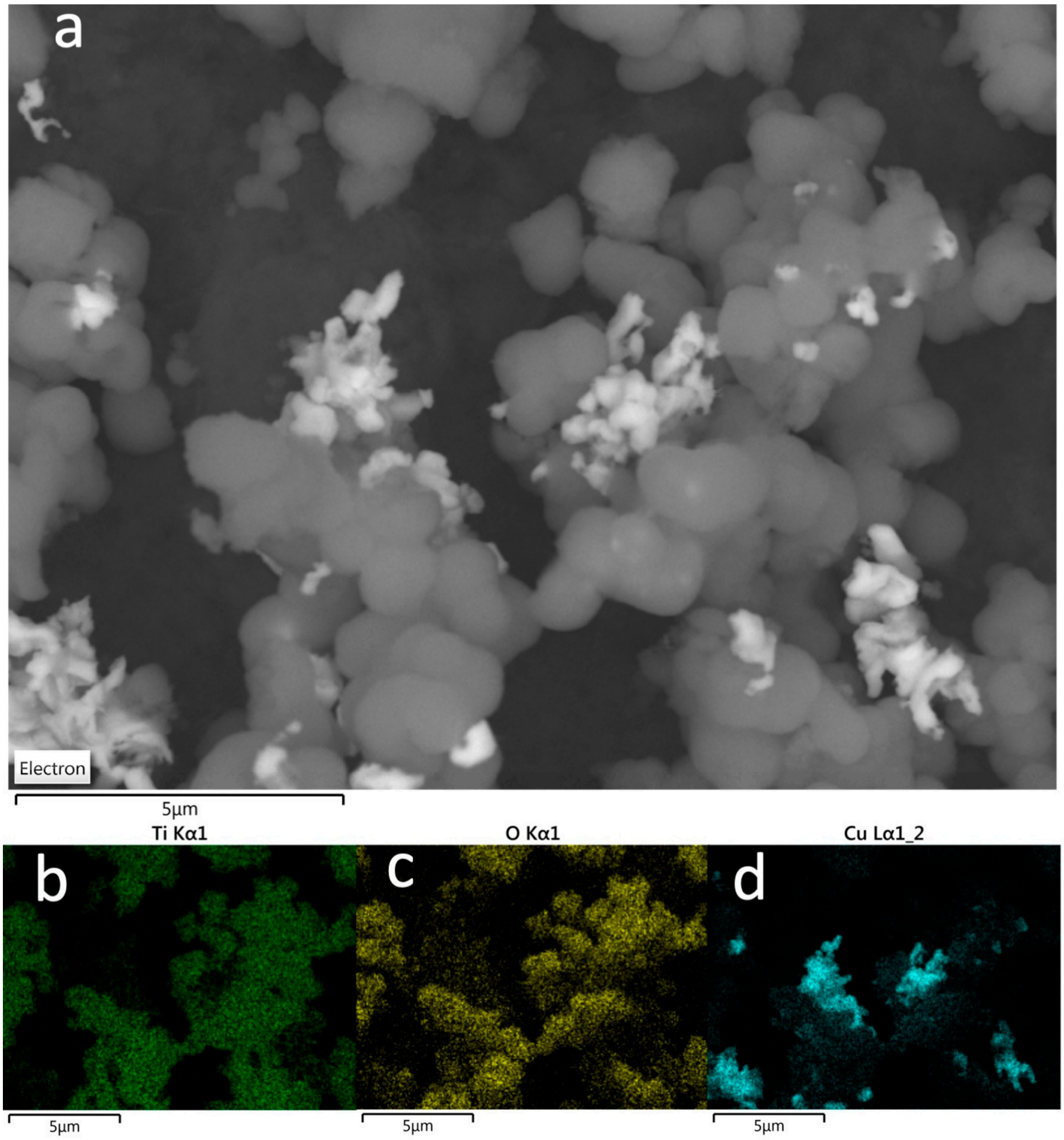

Figure S2. SEM micrograph of $1Cu/TiO_2$ (a) and EDX-mapping (b) Ti, (c) O and (d) Cu

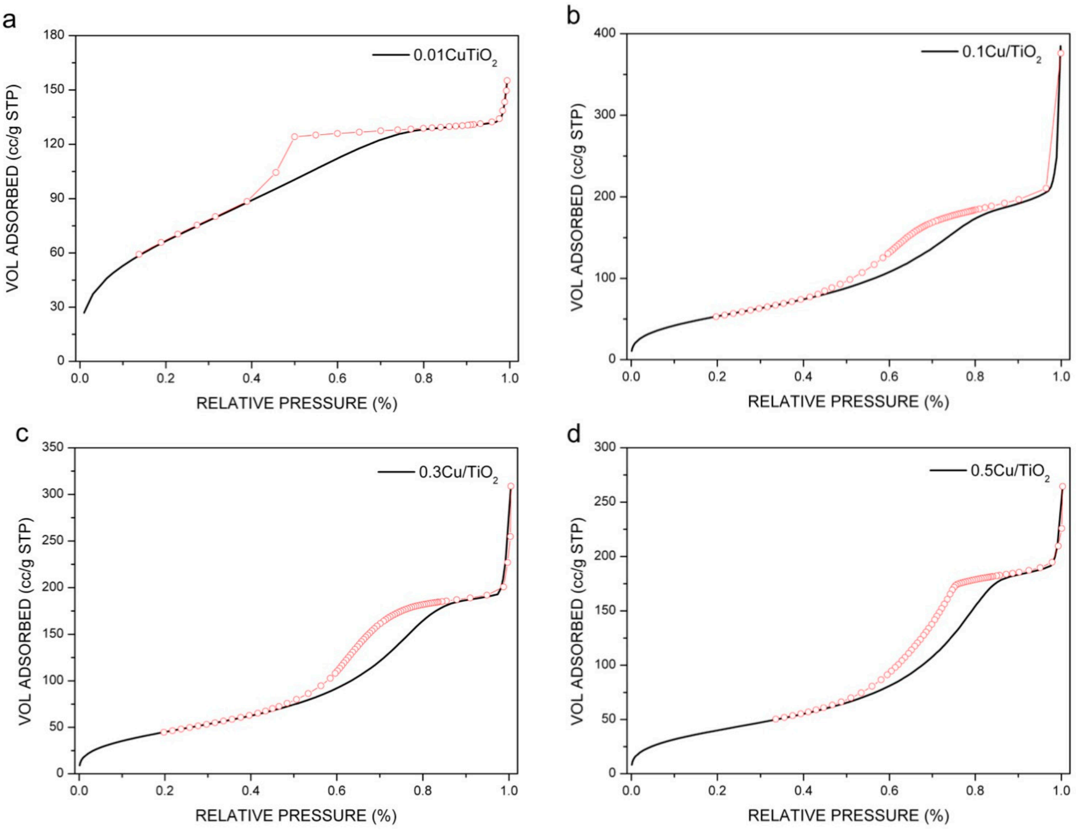

Figure S3. N_2 adsorption-desorption isotherm for (a) 0.01, (b) 0.1, (c) 0.3 and (d) 0.5 of copper content in Cu/TiO_2 samples.

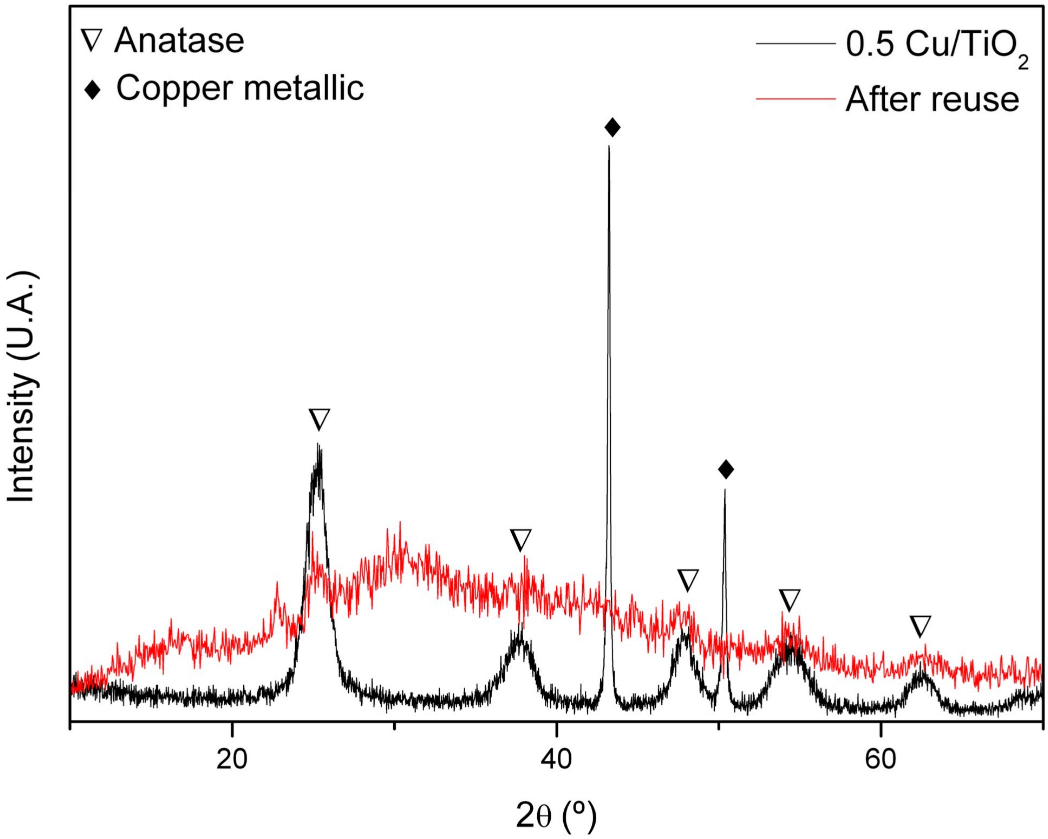

Figure S4. DRX pattern for $0.5CuTiO_2$ catalyst before (black) and after reuses (red), the catalyst is identified as mixture of anatase structure (TiO_2) and Copper metallic (Cu). After reuses the catalyst pattern is modified, is necessary to mention the catalyst recovery was very poor.
